# Epidemic Risk after Disasters

**DOI:** 10.3201/eid1209.060500

**Published:** 2006-09

**Authors:** John Watson, Michelle Gayer, Maire A. Connolly

**Affiliations:** * World Health Organization, Geneva, Switzerland

**Keywords:** communicable disease, risk assessment, letter

**To the Editor:** We conduct communicable disease risk assessments after humanitarian emergencies, including natural disasters, and would like to clarify the findings of Floret et al. (*1*) regarding the risk for epidemics in certain disaster settings. Natural disasters that do not result in population displacement, regardless of type of disaster, are rarely associated with increased risk for epidemics. However, large-scale population displacement, with consequent overcrowding in temporary settlements and disruption of water supply and sanitation, are indeed associated with increased risks for communicable disease transmission. This distinction is well documented (*2**–**4*). Increased communicable disease incidence after flooding and cyclones has been particularly well described (*5**,**6*). In addition, after a disaster of any type, epidemics may go undetected because of poor surveillance or because baseline surveillance data for diseases (such as dengue fever or malaria) are unavailable.

Although we agree with the authors that media reports are often exaggerated and that the risk for epidemics after certain types of natural disasters (e.g., volcanic eruption) is low, we believe the findings are somewhat misleading. Postdisaster communicable disease incidence is related more closely to the characteristics of the displaced population (size, health status, living conditions) than to the precipitating event.
