# Supplementary material for: Epidemic Risk after Disasters
Source: Emerg Infect Dis. 2006 Sep;12(9):1468–9. doi: 10.3201/eid1209.060500 (PMC3298287; doi:10.3201/eid1209.060500)
Supplement: Related texts

# Reply

**DOI:** 10.3201/eid1209.060665

**Authors:** Renaud Piarroux, Nathalie Floret, Jean-François Viel, Frédéric Mauny, Bruno Hoen

**Affiliations:** * University Hospital of Besançon, Besançon, France;; † University of Franche-Comté, Besançon, France

**In response:** Watson et al. stressed some points that may be important determinants in assessing the risk for epidemics following natural disasters (*1*). We agree that large-scale population displacement, with overcrowding and water disruption, is clearly a risk factor for disease transmission. This factor was probably the main cause of the measles and diarrhea outbreaks that occurred in the temporary settlements created after the eruption of Mount Pinatubo in the Philippines, as mentioned in our previous article (*2*). However, by studying >600 geophysical disasters (earthquakes, volcano eruptions, and tsunamis) that occurred in the last 20 years, we found that deleterious conditions such as large-scale population displacement with overcrowding and water disruption were uncommon and that epidemics were the exception, not the rule. We agree that some epidemics, especially if they are limited and develop well after the disaster, may remain undetected, as was discussed in our paper (*1*).

However, we do not concur with the opinion expressed by Watson et al. that the incidence of postdisaster infectious diseases is more related to the characteristics of the displaced population than to the precipitating event. Our findings are just the opposite. In contrast to the situation seen with flooding and cyclones, which are sometimes followed by outbreaks of waterborne diseases, such as cholera or leptospirosis, and vectorborne diseases (*3**–**6*), the study we carried out on geophysical disasters did not detect any notable outbreak except for the above-mentioned measles outbreak. Watson et al. illustrated their statement by referring to outbreaks following floods and hurricanes, and not earthquakes, tsunamis, or volcano eruptions. Further work must be carried out on epidemics after floods provoked by heavy rains and hurricanes.

## References

[1] Watson J, Gayer M, Connolly MA. Epidemic risk after disasters. Emerg Infect Dis. 2006;12:1468.1707311110.3201/eid1209.060500PMC3298287

[2] Floret N, Viel JF, Mauny F, Hoen B, Piarroux R. Negligible risk of epidemics after geophysical disasters. Emerg Infect Dis. 2006;12:543–7.1670479910.3201/eid1204.051569PMC3294713

[3] Beach M. China's problems persist after the flood. Lancet. 1998;352:1203. 10.1016/S0140-6736(05)60545-49777850

[4] Siddique AK, Islam Q, Akram K, Mazumder Y, Mitra A, Eusof A. Cholera epidemic and natural disasters; where is the link. Trop Geogr Med. 1989;41:377–82.2635456

[5] Sehgal SC, Sugunan AP, Vijayachari P. Outbreak of leptospirosis after the cyclone in Orissa. Natl Med J India. 2002;15:22–3.11855587

[6] Githeko AK, Lindsay SW, Confalonieri UE, Patz JA. Climate change and vector-borne diseases: a regional analysis. Bull World Health Organ. 2000;78:1136–47.11019462PMC2560843
